# *Piriformospora indica* and *Azotobacter chroococcum* Consortium Facilitates Higher Acquisition of N, P with Improved Carbon Allocation and Enhanced Plant Growth in *Oryza sativa*

**DOI:** 10.3390/jof8050453

**Published:** 2022-04-27

**Authors:** Prasun Bandyopadhyay, Bal Govind Yadav, Srinivasan Ganesh Kumar, Rahul Kumar, Karl-Heinz Kogel, Shashi Kumar

**Affiliations:** 1 International Centre for Genetic Engineering and Biotechnology, Aruna Asaf Ali Marg, New Delhi 110067, India; prasun.banerjee1986@gmail.com (P.B.); balgovind@icgeb.res.in (B.G.Y.); srinivasan@icgeb.res.in (S.G.K.); rahulkumar@icgeb.res.in (R.K.); 2 Institute for Phytopathology, Justus Liebig University, Heinrich-Buff-Ring 26, D-35392 Giessen, Germany; karl-heinz.kogel@agrar.uni-giessen.de

**Keywords:** arbuscular mycorrhizal fungi (AMF), endophyte, inoculation, microbes, plant growth-promoting rhizobacteria (PGPR), enzyme, protein

## Abstract

The soil microbiome contributes to nutrient acquisition and plant adaptation to numerous biotic and abiotic stresses. Numerous studies have been conducted over the past decade showing that plants take up nutrients better when associated with fungi and additional beneficial bacteria that promote plant growth, but the mechanisms by which the plant host benefits from this tripartite association are not yet fully understood. In this article, we report on a synergistic interaction between rice (*Oryza sativa*), *Piriformospora indica* (an endophytic fungus colonizing the rice roots), and *Azotobacter chroococcum* strain W5, a free-living nitrogen-fixing bacterium. On the basis of mRNA expression analysis and enzymatic activity, we found that co-inoculation of plant roots with the fungus and the rhizobacterium leads to enhanced plant growth and improved nutrient uptake compared to inoculation with either of the two microbes individually. Proteome analysis of *O. sativa* further revealed that proteins involved in nitrogen and phosphorus metabolism are upregulated and improve nitrogen and phosphate uptake. Our results also show that *A. chroococcum* supports colonization of rice roots by *P. indica*, and consequentially, the plants are more resistant to biotic stress upon co-colonization. Our research provides detailed insights into the mechanisms by which microbial partners synergistically promote each other in the interaction while being associated with the host plant.

## 1. Introduction

The interaction of plants with microorganisms co-evolved during evolution of land plants and resulted in stable symbiotic relationships, in which all partners benefit from the exchange of limited resources [1]. Ever since land plants have evolved, they have developed strategies by associating with microbes to evade abiotic and biotic stress. Mycorrhizal fungi and bacterial symbionts, such as plant-growth-promoting rhizobacteria (PGPR), colonize the majority of land plants. They improve plant productivity by enhancing the absorption range of roots for nutrients and by mechanisms that involve regulation of plant hormones such as auxin, ACC deaminase, cytokinin and gibberellin [2,3]. In turn, microbes rely upon plants’ carbohydrates for their growth. As a part of the rhizosphere community, mycorrhizal fungi are often associated with bacteria [4,5]. They either interact physically or become metabolically associated with the mycorrhizal life cycle. Deveau et al. [6] reported that mycorrhizal fungi show strain-specific interaction with bacteria influencing their formation and function. Hence, mycorrhizal fungi and rhizobacteria in combination improved seedling recruitment and plant nutrient acquisition [7].

In the rhizosphere, many bacteria have an undesirable effect on the neighboring microbial flora [8]. On the other hand, there are several reports that suggest that bacteria are required for the development and beneficial activity of mycorrhizal fungi [9], often acting as helper bacteria [8]. These PGPR are proficient in enhancing plant growth, and contribute towards plant resistance to fungal pathogens by producing siderophores, polyamines, auxins, antibiotic substances, and enzymes such as ACC-deaminase, chitinases, and histone acetylases [10,11,12]. Bacteria associated with the hyphae can promote their development and root colonization through supplying vitamins, phosphorus (P), sugars, nitrogen (N), and secondary metabolites to the fungal partner, or by acting as regulators of ATP molecules [13,14]. Mycorrhizal fungi produce extraradical hyphal networks in the soil, releasing carbon that in turn serves as an energy source for bacteria [15], further recruiting soil bacteria that colonize the hyphae [16]. In addition, bacteria that inhibit fungal growth (e.g., by secreting antifungal compounds) have also been reported [17] to improve soil fertility, increase crop productivity, and control plant pathogens biologically, and understanding the behavior of different kinds of beneficial microorganisms under association is therefore of prime concern. This information can subsequently be used in bioengineering of the plant microbiome and their management [18].

*Piriformospora indica* (*P. indica* syn. *Serendipita indica*) is a root-colonizing fungal endophyte, equally competent as arbuscular mycorrhizal fungi (AMF), with the additional advantage over AMF of being cultured axenically [19]. *P*. *indica* has the ability to enhance growth in plants and provide resistance against abiotic and biotic stresses [20] (Gill et al., 2016). The fungus activates induced systemic resistance (ISR) towards microbial pathogens [21], enhances antioxidants production in the plant tissues [22], mobilizes nutrients, and manipulates the hormonal balance of the plant [23,24,25]. Interestingly, *P. indica* has also been reported to host an endobacterium, characterized as *Rhizobium radiobacter* F4 [26], which on being isolated from the fungus shows properties for promoting growth in plants, although the causal mechanism has yet not been fully understood [27].

The *Azotobacter* genus contains free-living, nitrogen-fixing bacteria; unlike the *Rhizobium* species, they do not usually form symbiotic relationships with plants, however some *Azotobacter* species do tend to remain in close association with plants. *Azotobacter chroococcum* was the first species to be discovered in the genus *Azotobacter*. Since its discovery, *A. chroococcum* has been extensively studied in a number of research works for its role in plant nutrition and soil fertility. The W5 strain of the soil-dwelling heterotrophic N_2_-fixing Gram-negative gammaproteobacterium *A. chroococcum* has stimulatory effects on *P. indica*, and enhances mycelial growth and fungal sporulation. Metabolites produced by W5 have stimulatory effects, possibly by increasing the flux of carbon and nitrogen inside the fungal cells [28,29]. Some rhizobacteria also help AMF by improving the phosphate availability, which further helps in hyphal growth and development [30]. Co-inoculation of *A. chroococcum* along with *P. indica* in wheat under zinc deficiency has been shown to result in higher root and shoot biomass [31], while in *Artemesia annua*, the co-inoculants demonstrated significant increases in plant growth, photosynthetic pigments, total soluble sugar, soluble proteins, and flavonoids content [32]. Additionally, *P. indica* and *A. chroococcum* have been reported to increase growth and productivity, as well as improving mineral nutrition in various crop plants [20,33,34]. However, reports studying the underlying mechanism as well as the effect of co-inoculation at molecular level are still limited.

Previous reports on *P. indica* and *A. chroococcum* co-inoculation in plants helped us to understand the nature of interaction between these two microbes and their effect on plant growth. However, to understand the molecular mechanism of this interaction, studying the changes in plant enzymes, mRNA, and protein expression can give a comprehensive knowledge. The objective of understanding the role of free-living rhizobacteria on stabilizing mycorrhizal plant symbiosis enables us to exploit the relationships of the microbes in symbiosis. We therefore studied and herein report on the interaction of *P. indica* with *A. chroococcum* strain W5 within the root system of *Oryza sativa*. We found that *A. chroococcum* modulates the growth and sporulation of *P. indica.* Enzyme assay and leveraged label-free proteome analysis was conducted in order to investigate the impact of *A. chroococcum* on the *P. indica*-*O. sativa* proteome during the tripartite interaction. By combining proteomics data with the phenotypic analyses, we provide evidence that *A. chroococcum* increases the fitness of *P. indica* and improves its cooperation with *O. sativa.*

## 2. Materials and Methods

### 2.1. Selection of an Azotobacter chroococcum Strain for Interaction with Oryza sativa in Presence of Piriformospora indica

To study the interaction among *P. indica*, *A. chroococcum*, and *O. sativa*, we used two different strains of *A. chroococcum*: strain W5, which was originally isolated from the rhizosphere of wheat, and strain A41, which was received from the Department of Microbiology, Indian Agricultural Research Institute (IARI), New Delhi, India. The *P. indica* strain was gifted by Dr. Narendra Tuteja (International Centre for Genetic Engineering and Biotechnology (ICGEB), New Delhi, India), and was grown and maintained on Hill and Kaefer medium [35]. To examine the respective interactions of W5 and A41 with *P. indica*, plates containing Hill and Kaefer medium and agar were first inoculated in the center, with the fungal agar plug (5 mm) obtained from a freshly grown culture of *P. indica* and incubated at 28 °C in dark conditions. After two days of incubation, the two *A. chroococcum* strains were streaked separately on each plate’s periphery. The effect of the bacterial strains was determined by comparing the radial growth pattern and the spore yield of *P. indica*.

### 2.2. Inoculation of O. sativa Seedlings with P. indica and W5

Rice seeds (Pusa basmati-1, IARI) were surface-sterilized by rinsing for 1 min in 70% ethanol, followed by soaking in 4% NaClO solution for 20 min, then six subsequent washes with sterile water to remove any traces of chemicals left. Stratified seeds were germinated on water-agar plates (0.8%) at 25 °C in the dark for three days. Seedlings of the same age were planted in pots (soil + vermiculite) for greenhouse studies.

In controlled growth chambers, seedlings were further grown for a week in autoclaved sand with 75–80% relative humidity, day and night temperatures at 32 °C and 22 °C, respectively, and 16 h/8 h (light/dark) photoperiods with a light intensity of 250 µmol m^−2^ s^−1^. For inoculation, seedlings of the same age were inoculated with chlamydospore (5 × 10^6^ spores/mL) from freshly grown *P. indica* culture and W5 (OD~0.2) either in isolation or in combination, and were further transplanted into plastic pots (five seedlings per pot), which were filled with a sterile mixture of sand and vermiculite in a ratio of 2:1 by weight, and maintained in a greenhouse. Controls were treated with sterile water instead of microbial inoculants. Weekly applications of Hoagland’s nutrient solution were given to plants, and growth was monitored. The effectiveness of each treatment was determined in three biological replicates.

### 2.3. Nutrient Acquisition in O. sativa under Dual Inoculation

Uptakes of nitrogen and phosphorous either in the presence or absence of microbial inoculants were monitored 96 h post inoculation (HPI), and were measured in relative terms of nitrogenase, nitrate reductase, and phosphatase activities. The harvested roots were crushed in liquid nitrogen with the help of a mortar and pestle. Next, 100 mg of the sample was homogenized in an extraction buffer containing 250 mM Tris-HCl (pH 7.8), 1 mM EDTA, 5 μM flavin adenine dinucleotide, 1 μM Na_2_MoO_4_, 3 mM dithiothreitol, 1% BSA, 12 mM β-mercaptoethanol, and 250 mM phenylmethylsulfonyl fluoride. To obtain crude enzymes from the homogenized samples, they were centrifuged at 12,000 rpm for 20 min at 4 °C, after which the supernatant was used for enzyme assays. The Bradford (1976) method was used to measure soluble proteins. For each experimental set, biological replicates in three sets and three technical replicates were performed. Nitrate reductase assay was carried out according to Berger et al. [36], with the slight modification of quantification of NO^2−^ released during reaction in the assay mixture comprising the crude enzyme in 0.2 M HEPES, at a pH of 7.0, 15 mM KNO_3_, and 250 μM of NADH. To stop the reaction, samples were boiled for 3 min at 100 °C after 30 min had elapsed. The nitrite produced during the assay was measured using 1% sulphanilamide in 1 M HCl and 0.01% NEDD in water. By measuring the absorbance at 540 nm of the reaction mixture, the concentration of NO^2−^ was determined.

A few modifications were made to the method described by Fellbaum et al. [37] for the urease assay: 20 μL of crude enzyme and 50 μL of 100 nM Urea was added and incubated for 30 min at 37 °C. After diluting 20 μL of the reaction mixture with 980 μL of sterile distilled water, 100 μL of phenol nitroprusside and 200 μL of hypochlorite solution were added to detect and stop the reaction. Urease activity was determined spectrophotometrically at 636 nm.

Phosphatase activity was determined according to Khade et al. [38], with minor modifications. An aliquot of 20 μL of crude enzyme was incubated with 500 μL of 15 mM p-nitrophenyl phosphate and 0.25 M sodium acetate (pH 6.0) for 1 h. The reaction was terminated by adding 250 nM NaOH. The activity was determined spectrophotometrically at 412 nm.

### 2.4. Effect of W5 and P. indica on the O. sativa mRNA Expression Specific to Roots

After assessing the phenotypic and physiological responses of *O. sativa* in the presence of W5 and *P. indica* individually and in co-inoculation, three root-specific genes were selected that were putatively involved in lateral root/root hair development (root hair defective six-like class I; *RSL1*), nitrate transport (nitrate reductase 1; *NIA1*), and phosphate uptake (phosphate transporter 1; *PHT1*) (primer sequence in Supplementary Table S3).

Total RNA was isolated at 96 HPI with W5 and *P. indica* individually and in co-inoculation, and cDNA was synthesized using 1 µg RNA from each sample. For expression analysis, 20 ng cDNA was used as a template for quantitative PCR (qPCR) with gene-specific primers, and a plant-specific actin gene was used as internal control.

### 2.5. Impact of W5 on the P. indica-O. sativa Association

The effect of W5 on *P. indica*-*O. sativa* association was assessed by measuring the extent to which hexose was allocated to *P. indica* from plants in response to N and P transported to the plant through the fungus. To further validate the role of W5 on stabilizing the *P. indica-O. sativa* association, we quantified the fold difference in the expression of five selected fungal genes involved in bi-directional nutrient flux. Three selected genes—*Pi.UreA*, *Pi.GlutN*, and *Pi.GlutS*—participate in nitrogen transport, *Pi.HexT5* participates in hexose transport, and *Pi.PT* participates in phosphate transport (primer sequences in Table S3). Next, the roots of 96 HPI with W5 and *P. indica* individually and in co-inoculation were harvested for RNA isolation. To obtain cDNA from the isolated RNA, 1 µg of RNA from each treatment and control was used, and 20 ng cDNA was then used for expression analysis as template for qPCR with gene-specific primers and internal control (fungal *Tef α* gene).

### 2.6. Identification of Plant Root Proteins and Secretory Proteins during Fungal-Bacterial Co-Inoculation

For protein isolation, *O. sativa* plants grown in greenhouse after inoculation and control plants were used, roots of the plants were harvested, and total protein was isolated after 21 days of the interaction by following the protocol described in Raorane et al. [39]. For quantification of protein content, the Bradford method was used. For LC-MS/MS analysis sample preparation, analysis and data processing was conducted as follows:

### 2.7. Preparation of Sample for LC-MS/MS

Overall, 25 μL of protein samples were taken and reduced by using 5 mM tris 2-carboxyethyl phosphine (TCEP), which was further alkylated using 50 mM iodoacetamide and then digested with trypsin (1:50, Trypsin/lysate ratio) for 16 h at 37 °C. For cleaning the digested sample, a C18 silica cartridge was used to remove salt, while, samples were dried by using speed vacuum. We resuspended the pellet in buffer A (5% acetonitrile, 0.1% formic acid) after vacuum drying.

### 2.8. Mass Spectrometric Analysis of Peptide Mixtures

An EASY-nLC 1000 system (Thermo Fisher Scientific, Waltham, MA, USA) connected to a Thermo Fisher-Q Exactive with a nanoelectrospray ion source was used for all experiments. By using 1.8 µm of a C18-resin-filled 25 cm PicoFrit column with a dimension of 360 µm outer diameter, 75 µm inner diameter, and 10 µm tip, one microgram of the peptide mixture was resolved. The loading of peptides was conducted using buffer A, and elution was performed using buffer B, containing 95% acetonitrile and 0.1% formic acid with a flow rate of 300 nL/min for 100 min. A data-dependent top10 method was used to dynamically choose the most abundant precursor ions from the survey scans for the acquisition of MS data.

### 2.9. Data Processing

The MS/MS data thus produced was processed using Thermo Fisher Scientific search engine Proteome Discoverer (v2.2) against the *O. sativa*, *P. indica*, and *A. chroococcum* proteome database. The precursor and fragment mass tolerances were set at 10 ppm and 0.5 Da, respectively, for sequence searches. The protease used for peptide generation, Trypsin/P, was specified, with the maximum missing cleavage value as two. Carbamidomethyl on cysteine was set as fixed modification while, for the variable modification, oxidation on Met and acetylation on protein N-terminal and phosphorylation on site (S, T, Y) were specified for database search. We set the threshold for protein false discovery rate and peptide spectrum match to 0.01. *p*-values for the significance were calculated by using customized python scripts following Li et al. [40]. Using UniProt accession IDs as identifiers, bioinformatics analyses for rice proteins was conducted using the database for annotation, visualization, and integrated discovery (DAVID) [41]. For fungal proteins, the FungiFun [42] database was used, and a Venn diagram and heat map were developed using customized R scripts.

### 2.10. Statistical Analysis

All graphs were created using customized scripts in R by using package ggplot2 [43], and statistical analysis was performed using JASP [44]. The significance of the data obtained was checked by using a *t*-test.

## 3. Results

### 3.1. Interaction of P. indica with A. chroococcum Strains W5 and A41

**Figure 1:**
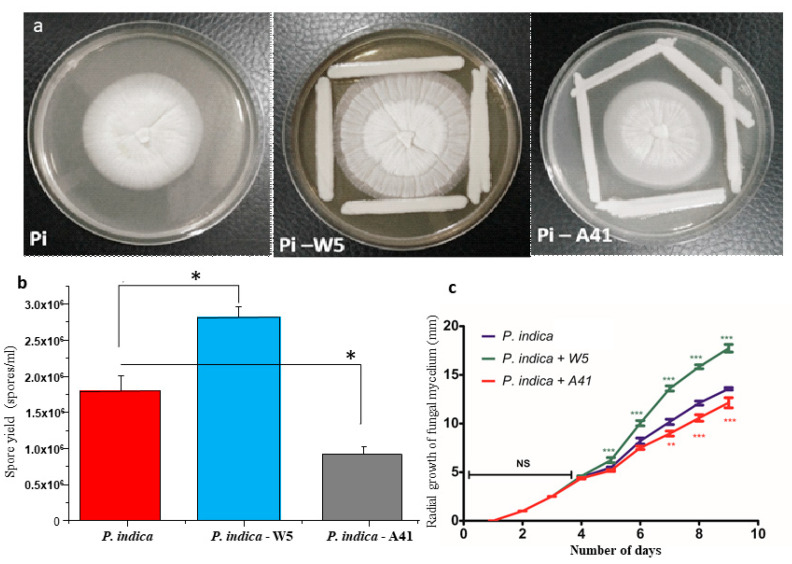
(**a**–**c**). Growth of *P. indica* was enhanced in the presence of *A. chroococcum* strain W5, while growth was observed to be inhibited in the presence of *A. chroococcum* strain A41. (**a**) Impact of two *A. chroococcum* strains on *P. indica* radial growth pattern. (**b**) Yield of chlamydospores in the presence of strains W5 and A41. (**c**) Radial growth of mycelium in the presence of strains W5 and A41. *P*-values indicate significant change in growth and spore yield in the presence of two strains (*n* = 3, *p*-value * ≤ 0.05, ** ≤ 0.01 and *** ≤ 0.001) compared to the control (*P. indica*). Standard error (SE) is represented by error bars, while NS represents ‘not significant’.

To identify bacterial strains with growth-promoting activity on *P. indica*, two *A. chroococcum* strains were confronted against the fungus on agar plates. After six days, the growth of *P. indica* was significantly stimulated by W5, whereas it was suppressed in the presence of A41 (unpaired two tailed *t*-test, *p* ≤ 0.05; Figure 1). It was concluded that W5 has growth-promoting activity, while A41 strain has growth-inhibiting activity. The W5 strain was further used as a stimulatory strain to study the interaction of the fungus and the bacterium in the rhizosphere of *O. sativa*.

### 3.2. Co-Inoculation Stimulates Phenotypic Traits in O. sativa

In order to comprehend whether W5 has a positive effect on the mutualistic association of *O. sativa* and *P. indica*, plant growth was measured both in the presence and absence of W5 after plants were inoculated with *P. indica*. At seven days post inoculation (DPI) with *P. indica* and W5 alone or in co-inoculation, there was an increase in the plant growth, primary root length, and lateral root density. The plant growth was measured with respect to the seedlings’ fresh weight, and the mean values of all the traits were compared to the control (Supplementary Figure S1b). The fresh weight (FW) of the seedling was significantly higher when *O. sativa* was inoculated with *P. indica* compared to the mock control (23.8%; unpaired *t*-test, *p* ≤ 0.05). When inoculated with W5 alone, the FW was 46.0% (significant: *p* ≤ 0.05) higher, whereas in co-inoculation it was 75.1% higher (significant: *p* ≤ 0.01) than the control seedlings. The results of the primary root length and lateral root density was similar to the FW, where co-inoculation improved the root formation in the developing seedling. Interestingly, plants inoculated with W5 had a higher lateral root density than plants inoculated with *P. indica*, and these values were comparable to the co-inoculation of plant roots with W5 and *P. indica* (Supplementary Figure S1c).

### 3.3. Co-Inoculation Contributes to Nutrient Acquisition of O. sativa

**Figure 2:**
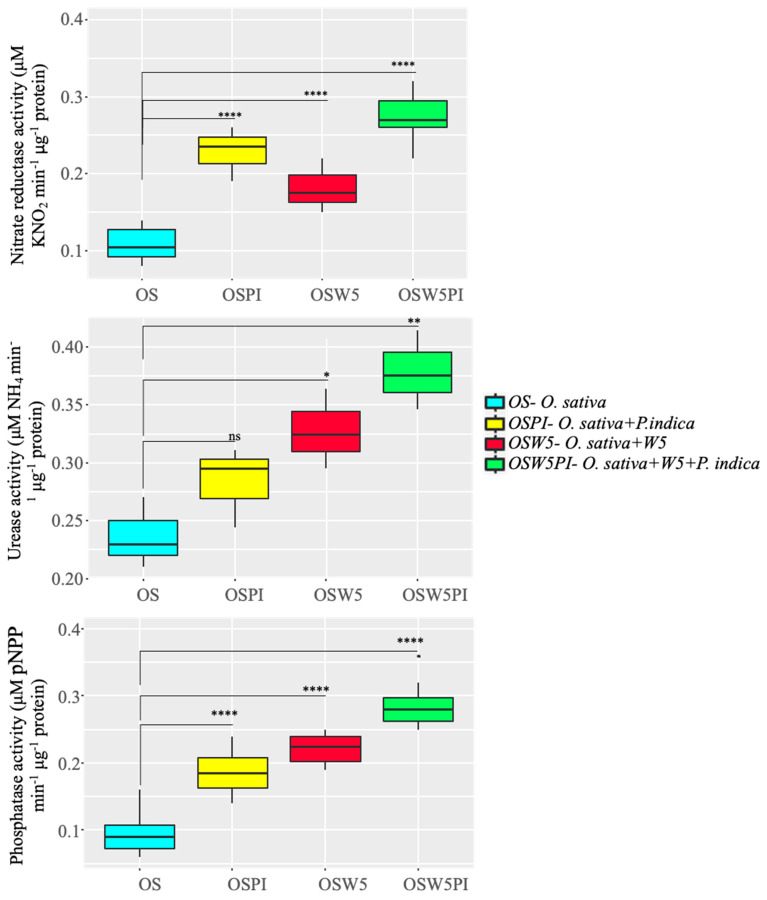
Impact of W5 and *P. indica* on nitrate reductase, urease, and phosphatase activity in *O. sativa* roots, after 96 HPI. Values represented as mean ± SE, *n* = 10, *p*-value (* ≤ 0.05, ** ≤ 0.01 and **** ≤ 0.0001) compared with *O. sativa*.

After inoculating *O. sativa* with *P. indica* chlamydospores (5 × 10^6^ spores/mL) and W5 (OD = 0.2), the physiological status of *O. sativa* was evaluated by measuring its nutrition acquisition ability. We specifically focused on N and P, as these are the macronutrients that have a directly role in the growth, development, physiology, and metabolism of the plants. Nitrate reductase activity measured 96 HPI was stimulated both in the presence of W5 (1.6-fold increase) and *P. indica* (2.1-fold increase), whereas in co-inoculation, the enzyme activity was more than 2.5-fold higher compared to the mock control (Figure 2).

A similar result was obtained for the urease activity that was enhanced in the presence of respective inoculants, whereas W5 was found to be more potent in enhancing the enzyme activity as it was 1.4-fold higher than the control vs. *P. indica* (1.2-fold higher). In co-inoculation, the influence of the microbes was alike to the nitrate reductase, with nearly a 1.6-fold increase in activity of the urease. These results support the hypothesis that in co-inoculation experiments, nitrogen is more easily acquired by the plant in comparison to the seedlings grown under single inoculation or without any microbial inoculant. A similar trend was observed for the phosphatase activity, as phosphatase activity was higher in the presence of W5 (2.3-fold) compared to mock control or inoculation with *P. indica*, where the phosphatase activity was 1.9-fold higher than the control. Upon co-inoculation, the phosphatase activity was enhanced and highly significant (*p*-value < 0.0001), with a nearly 3-fold increase in the accumulation of phosphate (Figure 2).

### 3.4. Impact of W5 and P. indica on the Selected O. sativa RNA Transcripts

**Figure 3:**
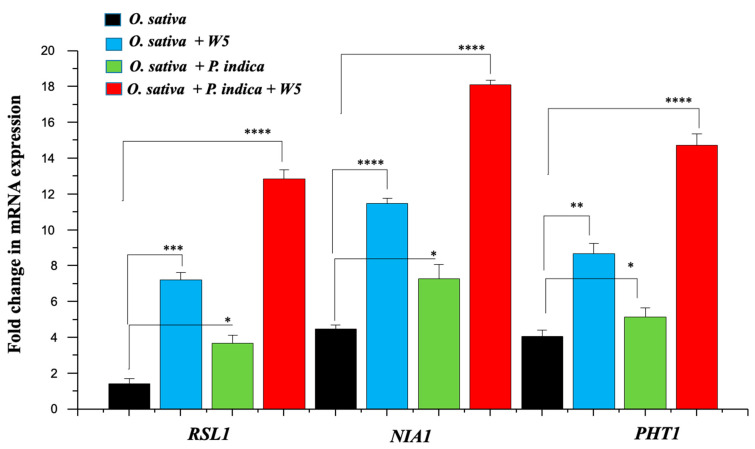
Changes in the expression of selected *O. sativa* genes *RSL1, NIA1*, and *PHT1* observed 96 HPI with *P. indica* and W5 individually or in co-inoculation. Values represented as mean ± se. (*n* = 3, *p*-value * ≤ 0.05, ** ≤ 0.01, *** ≤ 0.001 and **** ≤ 0.0001) compared with control *(P. indica*). Standard error (SE) is represented by error bars.

After 96 HPI, significant (*p* value ≤ 0.05, unpaired *t* test) changes were observed in all the selected plant genes used to check the influence of the endophyte and PGPR on root growth and nutrient uptake. *RSL1* is a basic helix-loop-helix transcription factor expressed in root hair cell initials of the root meristem, where it positively regulates root hair cell development. In the presence of W5, transcript levels of *RSL1* were found to be higher by 5.1-fold, whereas in the presence of *P. indica*, the fold change value was less (2.6-fold) and was comparable to that of the control (Figure 3). However, in co-inoculation, the fold change was 9.1-fold in comparison to the control. This correlates to the phenotypic responses observed in the presence of the respective inoculants. The *NIA1* that encodes the enzyme nitrate reductase was upregulated by 2.6-fold by W5, and only 1.6-fold by *P. indica*, in comparison to the mock control. Upon co-inoculation, the transcript fold change was much higher, with a 4-fold increase in comparison to the control. These results clearly indicate that W5 and *P. indica* induce higher assimilation of nitrite, as in the case of nitrate reductase assay. Indistinguishably to *NIA1*, *PHT1*, which encodes a family of phosphate (Pi) transporters that mediate phosphorus uptake and re-mobilization in plants, was 2.1-fold upregulated by W5 when compared to the control. The transcript level in this case was higher compared to that induced by *P. indica* (1.3-fold). Based on these results, we conclude that W5 has better affinity for solubilizing and mobilizing phosphate in comparison to *P. indica*. However, with co-inoculation, induction in *PHT1* transcripts was much higher, with a 3.6-fold change in comparison to the control (Figure 3). The results of the expression analyses, like the enzyme assays, clearly indicated that co-inoculation results in higher nutrient acquisition from the soil and a better physiological response and growth in the plant.

### 3.5. W5 Stimulates Carbon (C), Nitrogen (N) and Phosphorous (P) Allocation by P. indica into O. sativa

An invertase assay at 96 HPI revealed higher hexose allocation in the roots co-inoculated with W5 and *P. indica* (*p*-value ≤ 0.05) in comparison to the roots inoculated with either W5 or *P. indica* alone (Supplementary Figure S2). We measured the expression of *Pi.GlutS* involved in nitrogen transport, *Pi.UreA* involved in arginine breakdown, *Pi.HexT5* involved in hexose transport, and *Pi.PT* involved in phosphate transport, to study the change in patterns of nitrogen and phosphorous acquisition. *Pi.HexT5* was significantly upregulated by W5 in comparison to *P. indica* (2.1-fold higher). Our result was similar to the observations made by Rani et al. [45].

**Figure 4:**
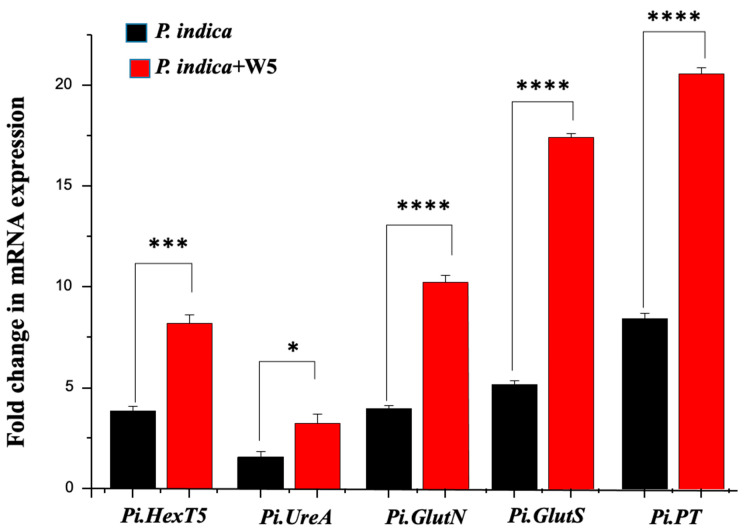
Relative fold changes of the *P. indica* genes *Pi.HexT*, *Pi.UreA*, *Pi.Glut.N*, *Pi.Glut.S*, and *PiPT* observed in-vivo within *O. sativa* roots 96 HPI with *P. indica* either in the presence or absence of W5. Values signifies the mean of replicates (*n* = 3, *p*-value * ≤ 0.05, *** ≤ 0.001 and **** *p* ≤ 0.0001) compared with *P. indica*. Standard error (SE) is represented by error bars.

In both conditions, phosphate- and nitrogen-responsive genes were found to be correlated with the hexose allocation. In the case of co-inoculation with W5, the nitrogen responsive genes *Pi.UreA, Pi.Glut.N*, and *Pi.Glut.S* were 2-fold, 2.6-fold, and 3.4-fold upregulated, respectively, and *Pi.PT* was 2.4-fold upregulated. However, in plants inoculated with *P. indica* only, the fold change values were significantly reduced (Figure 4). *Pi.HexT5* transcript level was increased to 2.1-fold in the presence of W5 as compared to *P. indica* only. Thus, higher nitrogen and phosphate was transported to the plant, and higher carbon was allocated to the fungus from the plant roots. These results are substantive to conclude that W5 has a positive bearing on the mutualistic relationship of *P. indica* with *O. sativa*.

### 3.6. Identification of Differentially Abundant (DAP) Proteins in Individual and Co-Inoculation with P. indica and W5

**Figure 5:**
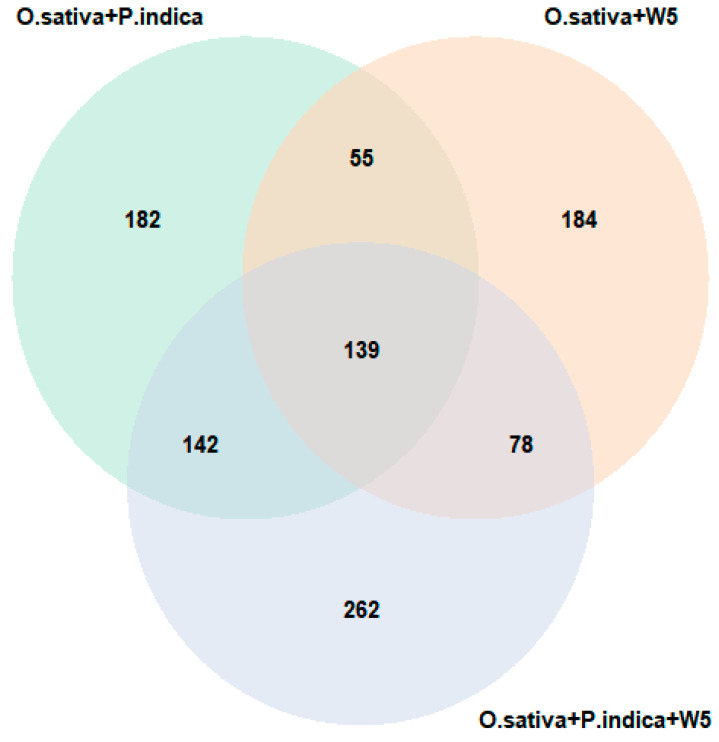
Venn diagram showing significantly (*p*-value, *p* ≤ 0.05) differentially abundant proteins that were identified in *O. sativa* under three different conditions of plant microbial interactions.

**Figure 6:**
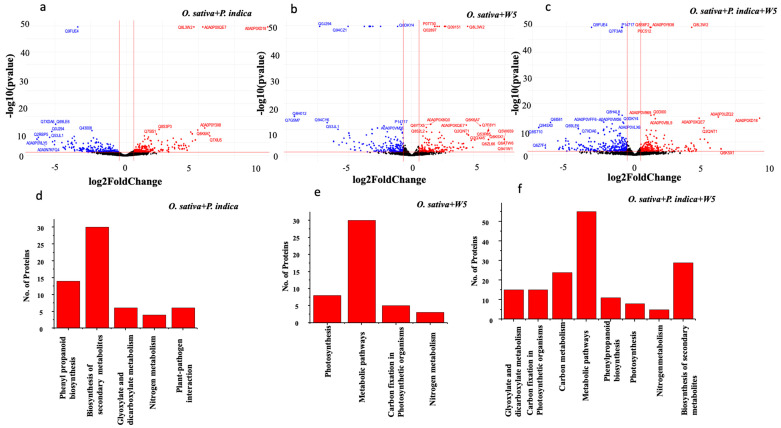
Significantly differentially abundant proteins with 0.5-fold up and down regulation and KEGG pathway enrichment. (**a**) Volcano plot for the differentially abundant proteins identified in *O. sativa* when inoculated with *P. indica* after 21 days of interaction. Proteins that were significant (*p*-value < 0.05) and upregulated are indicated by the color red, significantly downregulated proteins are indicate d by the color blue, and the color black indicates DAPs that were insignificant. (**b**) Volcano plot of DAPs in *O. sativa* inoculated with W5. (**c**) Volcano plot of DAPs in *O. sativa* co-inoculated with W5 and *P. indica.* (**d**–**f**) KEGG pathway enriched (*p*-value *p* < 0.05) by upregulated DAPs in *O. sativa* inoculated with *P. indica*, W5 alone, and in combination of both.

A total of 3511 *O. sativa* proteins (Supplementary Table S1) were identified at 21 DPI based on LC-MS analysis in all the three treatments. DAPs were identified using the *p*-value < 0.05 and log2fold-change (log2FC) equal to or more than 0.5, and equal to or less than −0.5, resulting in the identification of 527, 455 and 604 DAPs in *O. sativa* inoculated with *P. indica*, W5, or both, respectively (Supplementary Table S1). Among these, 182 were specific to *O. sativa* inoculated with *P. indica*, 184 were specific to *O. sativa* inoculated with W5, and in co-inoculation with *P. indica* and W5 a total of 262 specific DAPs were observed. A total of 139 DAPs were common for all three sets (Figure 5). Some DAPs that were identified in the different treatments showed very high *p*-values and a higher fold change value, as shown in volcano plots (Figure 6a–c). However, GO annotation for most of these are not available. One DAP exhibiting peroxidase activity was common to all the treatments with a *p*-value of 2 × 10^−50^. This finding was similar to previous reports on tobacco, where there was a transient increase in peroxidase in mycorrhizal roots [46]. The Venn diagram revealed that most DAPs were exclusively expressed in *O. sativa* co-inoculated with W5 and *P. indica*. This trend was consistent with our hypothesis that during co-inoculation there are higher levels of proteomic changes, as it involves the action of both the endophytic fungus and the PGPR.

Among these 517, 455 and 604 DAPs (Figure 6), those upregulated were further used for the KEGG (Kyoto Encyclopedia of Genes and Genomes) pathway enrichment analysis via database for annotation, visualization, and integrated discovery (DAVID), and as an identifier, UniProt accession IDs were used. Enrichment analysis was performed for each treatment individually. For *O. sativa* inoculated with *P. indica*, there were five pathways with a *p*-value less than 0.05 that were significantly enriched: biosynthesis of secondary metabolites, phenylpropanoid biosynthesis, glyoxylate and dicarboxylate metabolism, plant pathogen interaction, and nitrogen metabolism. For *O. sativa* inoculated with W5, four pathways were enriched significantly (*p*-value < 0.05), including metabolic pathways, nitrogen metabolism, photosynthesis, and carbon fixation. In plants co-inoculated with W5 and *P. indica*, eight pathways were significantly enriched, which included metabolic pathways (Figure 6d–f), biosynthesis of secondary metabolites, carbon fixation, carbon metabolism, glyoxylate and dicarboxylate metabolism, phenylpropanoid biosynthesis, and nitrogen metabolism (Supplementary Table S2).

We also performed enrichment analysis with the upregulated DAPs that were common to all three treatments. Five pathways that included nitrogen metabolism, carbon metabolism, lineloic acid metabolism, phenyl propanoid biosynthesis, and biosynthetic pathways of secondary metabolites (Supplementary Table S2), were significantly enriched by 139 DAPs that were shared by all the treatments (Supplementary Figure S3). Among the 66 upregulated DAPs that were common in all treatments, differential abundance of the proteins in co-inoculation was more prominent as compared to single inoculation with either *P. indica* or W5 alone, as the log2fold change was higher in the case of co-inoculation.

Furthermore, nitrogen metabolism, carbon fixation, and glyoxylate metabolism was also significantly enriched, and a similar observation was made in barley under salt stress in the presence of endophytic fungus [47]. Besides the proteins involved in nitrogen metabolism, many other proteins were also differentially expressed, including phosphoesterase, calcium-dependent protein kinases, pathogenesis-related proteins, peroxidases, multicopper oxidase, chitinase, aminotransferase, and o-methyltransferase. Among the differentially expressed proteins was Phosphoesterase, which is involved in phosphorous uptake from soil. In a recent study [48], it was reported that the Phosphoesterase family of proteins is upregulated in rice plants grown under phosphate-deficient soil. Multicopper oxidase was another DAP that is involved in phosphate metabolism; it has been reported to sense phosphate availability in root meristem and also plays a role in phosphate starvation [49]. Calcium-dependent protein kinases (CDPKs) are involved in regulating downstream components of calcium signaling, and an overexpression of CDPK in rice plant enhances the growth under low nitrogen conditions [50]. Pathogenesis-related proteins are known to play a crucial role during plant pathogen interactions and plant development, however, it has also been reported that overexpression of these proteins leads to higher root and shoot biomass, in addition to providing biotic and abiotic stress tolerance [51]. The activity of Chitinase, a protein that is involved in the degradation of chitin in plants, was found to be higher in the presence of endophytic fungus and PGPR in the common bean [52]. Another DAP called O-methyltransferase, which is involved in the regulation of melatonin synthesis, acts by regulating growth and development, and alleviates stress [53]. Serine glyoxylate aminotransferase, an enzyme that is involved in the transamination of glyoxylate into glycine, which is then converted into serine, was found to be upregulated. It has been reported that the overexpression of gene encoding in this protein results in enhanced efficiency of nitrate and ammonium assimilation in transgenic lines that show higher amino acid accumulation [54].

### 3.7. Identification of DAP Proteins in P. indica in Presence of W5

In order to identify fungal proteins specifically present in the colonized roots of *O. sativa* in the presence of W5, fungal proteins were isolated from colonized rice roots. DAPs were identified based on the log2foldchange values of their abundance proteins that showed abundance of 0.5 times or higher, as well as those showing an abundance score of −0.5 or less, and *p*-values less than or equal to 0.05. Based on these parameters, a total of 38 fungal proteins were identified, of which 12 were upregulated and 26 were downregulated. Enrichment analysis of these DAPs was performed using the FungiFun database. Overall, seventeen proteins out of the 38 were enriched in nine different gene ontology (GO) terms under the biological process (BP) and molecular function (MF) categories. Maximum GO enrichment was found to be the translational process in the BP category, followed by the oxidation reduction process. For the MF category, structural constituent of ribosome, peroxidase activity, and transaminase activities were the most enriched GO terms (Supplementary Figure S4). The upregulated proteins included aspartate aminotransferase, D-xylose reductase, NADPH dehydrogenase, aspartate protease, and haloacid dehalogenase. The DAPs that were downregulated included peroxidase, glutathione peroxidase, and Ketol-acid reductoisomerase. D-xylose reductase, which has a role in lignin degradation, was found to be downregulated in this study, as the endophytes fungal cells are in close association with the plant cells.

## 4. Discussion

### 4.1. Effect of Microbes on Plant Growth 

In the rhizosphere, interactions among the microbial community play very important role in plant growth promotion [29]. Individual inoculation of *A. chroococcum* strain W5 is involved in the improved growth of maize [55] and wheat [56]. Co-inoculation of W5 with *Trichoderma* resulted in improving plant growth and soil nutrient availability in chickpea [57], wheat, and cotton [58]. Co-inoculation of W5 and *P. indica* in *Stevia* has also been shown to result in enhanced plant growth, higher antioxidant potential, and increased steviol glycoside content [59]. *P. indica* along with rhizobacterium *Bacillus cereus* are known to help in rhizosheath formation, which improves water use in drying soil [60]. Recently, it has been shown that the use of PGPR and AMF in lettuce plants can give similar or increased yield, as it was obtained through the use of NPK fertilizers [61]. In this study, we have established bacterial strains influencing the radial growth of *P. indica*, indicating mutual interaction through secretory effectors. Previously, it has been shown that *A. chroococcum* W5 stimulates the growth of *P. indica* by activating the nitrogen uptake and metabolism [28]. Similarly, *A. vinelandii* strain SRIAz3 was also reported to promote spore formation and hyphal growth in *P. indica* [62]. Proteomic study results of *P. indica* in the presence of W5 revealed an overexpression of proteins that play an important role in oxidative stress during symbiosis, which is in good agreement with the results of Sędzielewska et al. [63] and Serrano-Bueno et al. [64].

### 4.2. Enzyme Activity and Expression Analysis of Nutrition Acquisition Genes during Individual Inoculation and Co-Inoculation in O. sativa

As we observed a change in the phenotypic attributes of *O. sativa* plants, we observed similar results in the enzymatic assay and expression analysis of genes involved in the nutrition acquisition process. In plants, nitrate reductase catalyzes the first step in reducing nitrate to organic forms within the plant, and a higher NR activity indicates that more and more nitrogen is being acquired by the plants in the presence of *P. indica* and *A. chroococcum*. A similar result was reported in *Acacia gerrardii* in the presence of mycorrhizal fungi and endophytic bacteria [65], while single inoculation of mycorrhiza in plants leads to higher nitrate reductase activity and better nitrogen uptake [15]. Enhanced urease activity and increased phosphatase activity indicated that in the presence of endophytic fungi and PGPR, plants can easily solubilize the nutrients available in the soil and utilize them. These results are in good agreement with those obtained by Mader et al. [66]. Results of mRNA expression analysis was similar to the results obtained for enzyme assays, which revealed that under fungal colonization, in the presence of PGPR as well as in co-inoculation, expression of *RSL1*, *NIA1*, and *PHT1* is increased, explaining the higher biomass, increased adventitious growth, and higher assimilation of nitrogen and phosphorous.

### 4.3. Proteome Changes in P. indica during Inoculation with W5

We also observed an upregulation of proteins in *P. indica*, which are mostly involved in the nitrogen metabolism and degradation of lignin, a component of cell wall of plants. GO enrichment analysis also indicated that W5 leads to upregulation of proteins involved in carbon and nitrogen metabolism in *P. indica*. The proteomic results were similar to the enzyme assays and mRNA expression analysis, validating our results. Based on this observation, we can conclude that W5 supports growth of *P. indica* and colonizing roots’ cortical cells, an observation previously made by Salvioli et al. [67] and Nizam et al. [68] in *Gigaspora margarita* and *P. indica*, respectively.

### 4.4. Proteomic Changes in O. sativa after Inoculation with W5 and P. indica and in Combination

Proteomic analysis of rice plants gave insights into the mechanism that possibly lies in the tripartite interaction of plant endophytic fungus and bacteria. Among the various proteins identified in these experiments and in all the treatments, we observed nitrogen metabolism to be the pathway that was commonly enriched, clearly indicating that during the tripartite interaction of plant and microbes, there is a higher assimilation of nitrogen in *O. sativa*. DAPs that were identified in this study were similar to our mRNA expression and enzyme assay, however, we could not identify a phosphate transporter protein that was abundantly present in the enzymatic assay and mRNA expression. On the other hand, we could identify other DAPs like *Phosphoesterase* and multi copper oxidase, which are responsible for higher assimilation of phosphate to the plant from the soil during inoculation, with *P. indica*, W5, and in combination. Another protein that could be associated with the higher root growth and biomass in rice plants is *O-methyltransferase*, a protein that is an essential component in the synthesis of melatonin, and as reported by Tan et al. [69], promotes root regeneration by stimulating the development of the root system.

### 4.5. Mechanism of Nutrient Uptake and for Increased Growth in O. sativa

**Figure 7:**
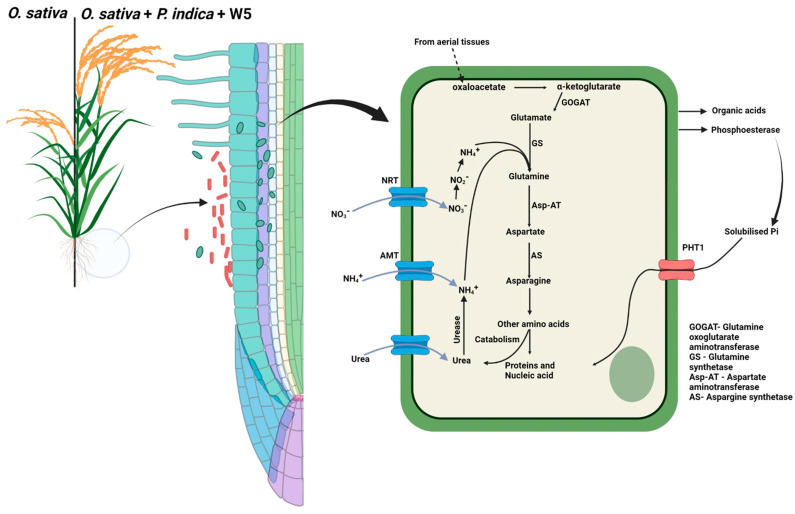
Hypothetical model for explaining the mechanism of nutrient uptake that promotes higher plant growth and biomass in the presence of endophytic fungus *P. indica* and plant growth-promoting rhizobacterium *A. chroococcum* strain W5.

The absorption of nitrogen, which is an essential mineral nutrient for the plants, takes place through the roots from the soil in various forms such as ammonium (NH_4_^+^) and nitrate (NO_3_^−^) ions [70]. We observed that after mycorrhization by fungi in rice plants, N is acquired by plant roots via active transport through the plasma membrane of epidermal and cortical cells. N is utilized by plants for a variety of metabolic processes, including the biosynthesis of amino acids by glutamine synthetase and glutamine synthetase (GS-GOGAT). It is further used for the synthesis of other amino acids by the activity of aminotransferase, and these amino acids are used in protein synthesis resulting in molecular and physiological changes. Urease, an enzyme that is involved in the degradation of urea into ammonia and carbon dioxide, plays a vital role in ammonia assimilation, as urea is produced inside the plant cell by catabolism of arginine. Another source of urea is rhizosphere, where urea is supplied mainly as a fertilizer and transported inside the cell by the action of membrane transporters. Similarly, phosphorous is solubilized and assimilated in plants through the action of organic acids and phosphoesterase secreted by plants, which then becomes upregulated in the presence of *P. indica* and *A. chroococcum*. Organic acids and phosphatases (here phosphoesterase) solubilize various phosphate containing substrates in the rhizosphere. Transporter proteins like *PHT1*, the expression of which is increased in the presence of endophyte *P. indica* and PGPR *A. chroococcum* strain W5, are involved in the transport of phosphate by roots from the external rhizosphere to the inside of the plant cell (Figure 7). Presence of inoculants in the soil also upregulate the expression of genes that in turn increases the root hair formation, resulting in enhanced absorption by the roots. These changes are brought by accelerating the production of hormones and metabolites [71], which are responsible for higher growth and biomass production. Thus, besides improving the nutritional uptake in plants, *A. chroococcum* also plays an important role in stabilizing the plant and endophyte association by solubilizing nutrients and promoting growth of endophytic fungi, leading to improved plant growth.

## 5. Conclusions and Future Perspectives

In conclusion, in the inoculation of *O. sativa* with endophytic fungus *P. indica* and PGPR *A. chroococcum* W5 individually, as well as in co-inoculation conditions, we observed enhanced growth and biomass at phenotypic level, higher enzymatic activities of certain enzymes involved in nutrient solubilization, and increased transcription for the genes involved in nutrient transport and metabolism. Our results based on the proteomic analyses indicate that there are protein level changes in rice plants in the presence of microbes that are specific to the pathways involved in nitrogen, carbon and glyoxylate metabolism. In the case of co-inoculation, changes were more pronounced than in single inoculations with either of these.

Findings of this research hold potential in improving the extent of nutrient acquisition by rice plants from the soil to enhance crop productivity, as extensive use of synthetic inorganic fertilizers and chemicals has only caused major harms to the microbial, animal, and plant biodiversity, as well as on the surrounding ecosystems [72,73]. However, before we start utilizing the findings of this research on a large scale, these results should be confirmed and validated through transgenic or genome editing technologies through CRISPAR-CAS. A comprehensive understanding of the relationship of plants with microbes can definitely be utilized to improve productivity and tolerance to biotic and abiotic stresses, and will benefit plants to better survive changing climatic conditions. Metabolic engineering of synergistic interactions between plants and microbes will not only improve the fitness of plants, but also reduce the chemical inputs leading to sustainable crop production worldwide.

## Data Availability

Supporting information and data related to experiment is provide in the supplementary information however, any further information related to data can be obtained by writing to first or corresponding author.

## Supplementary Materials

The following supporting information can be downloaded at: https://www.mdpi.com/article/10.3390/jof8050453/s1, Figure S1: Impact of W5 and *P. indica* on the growth of *O. sativa* (PB-01) tested individually and in co-inoculation; Figure S2: Effect of W5 and *P. indica* on invertase activity of *O. sativa* roots either individually or in co-inoculation compared with control (*O. sativa*). Figure S3: Heatmap showing different metabolic pathways significantly (*p* value < 0.05) enriched by 139 differentially abundant proteins (DAPs) present at all three-interactions (1. *O. sativa* + *P. indica;* 2. *O. sativa* + W5; 3. *O. sativa* + *P. indica +* W5). Figure S4: Pie chart showing significantly (*p* value, *p* ≤ 0.05) enriched biological processes and molecular functions for the genes corresponding to the *P. indica* DAPs, identified in the roots of the *O. sativa* in the presence of the W5. Table S1: List of total proteins identified in different groups in LC-MS/MS analysis along with their description, fold change value and *p*-values; Table S2: List of different groups enriched in KEGG pathway by upregulated DAPs in *O. sativa* inoculated with the *P. indica*, W5 alone and in combination of both. KEGG pathway enriched (*p* value *p* < 0.05) by upregulated DAPs in *O. sativa* inoculated with the *P. indica*, W5 alone and in combination of both; Table S3: List different primer pairs used in this study for expression analysis.
