# Supplementary material for: Piriformospora indica and Azotobacter chroococcum Consortium Facilitates Higher Acquisition of N, P with Improved Carbon Allocation and Enhanced Plant Growth in Oryza sativa
Source: J Fungi (Basel). 2022 Apr 27;8(5):453. doi: 10.3390/jof8050453 (PMC9146537; doi:10.3390/jof8050453)
Supplement: Supplementary file 1 [file jof-08-00453-s001.zip › Figures S1-S4.pdf]

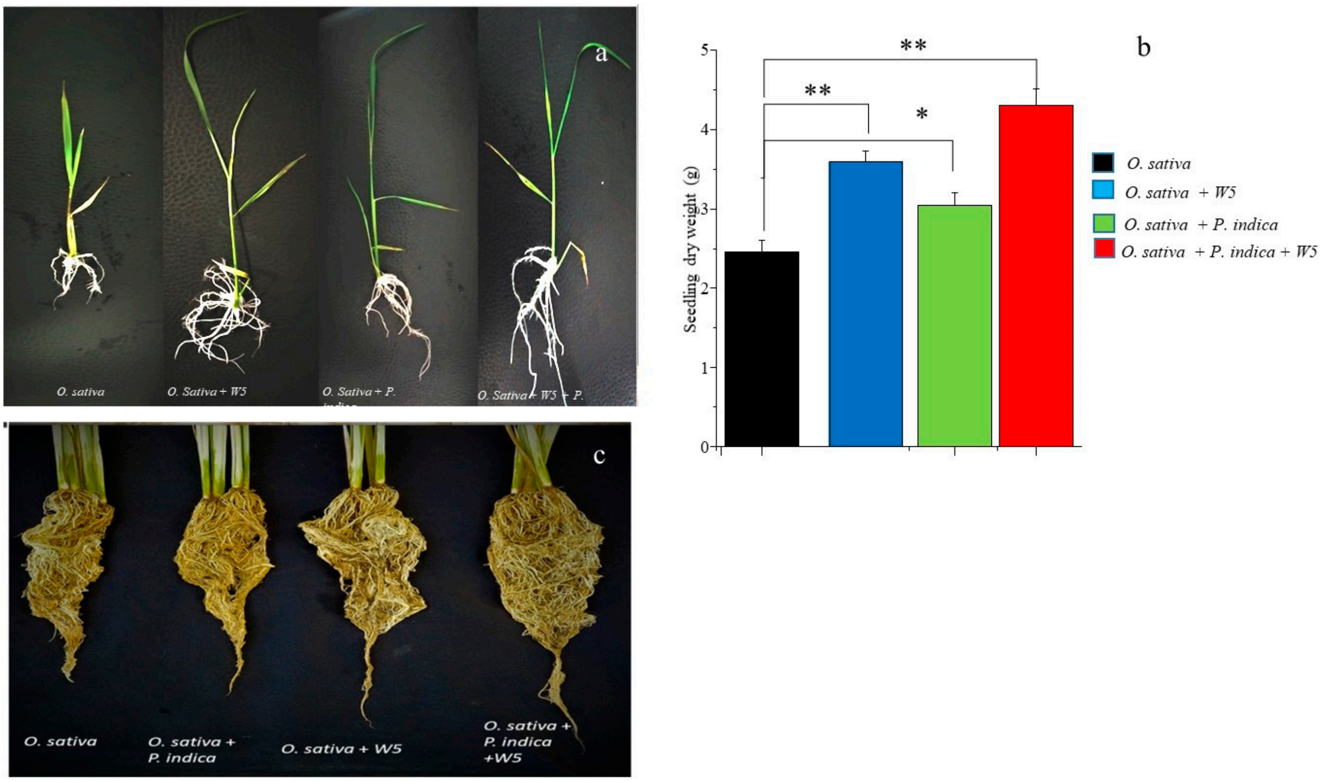

Supplementary Figure S1. a-c. Impact of W5 and *P. indica* on the growth of *O. sativa* (PB-01) tested individually and in co-inoculation. (a) The *O. sativa* seedlings under laboratory conditions after inoculating with the *P. indica* and W5 individually as well as in combination of both the organism. (b) Graph showing seedling fresh weight along with control (Pvalue * ≤ 0.05and **P≤0.01). Standard error (SE) is represented by error bars (c) Lateral roots density of control and inoculated plants with W5 and *P. indica*.

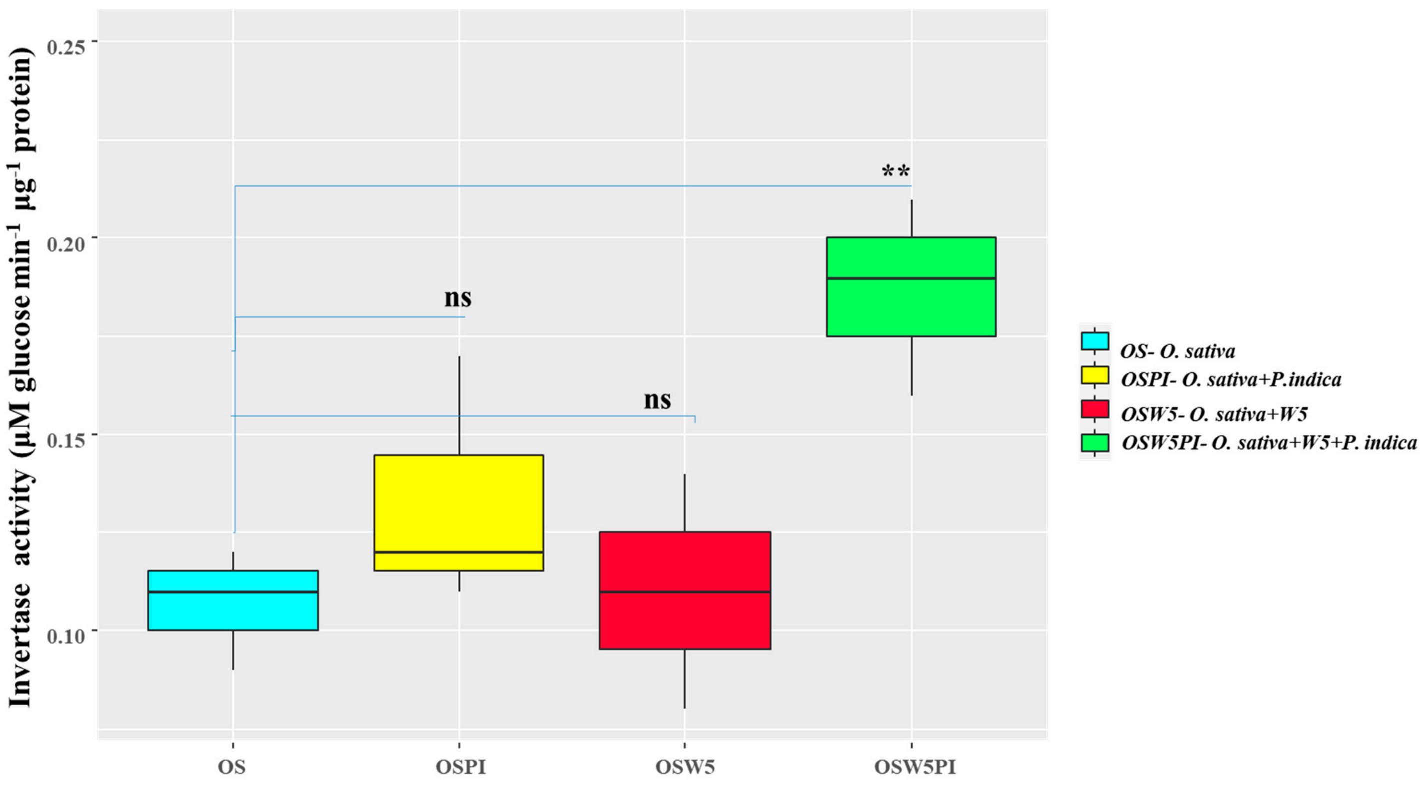

Supplementary Figure S2. Effect of W5 and *P. indica* on invertase activity of *O. sativa* roots either individually or in co-inoculation compared with control (*O. sativa*). Values signifies the mean ±se (n=10, Pvalue **≤0.01, ns- not significant) of raw values.

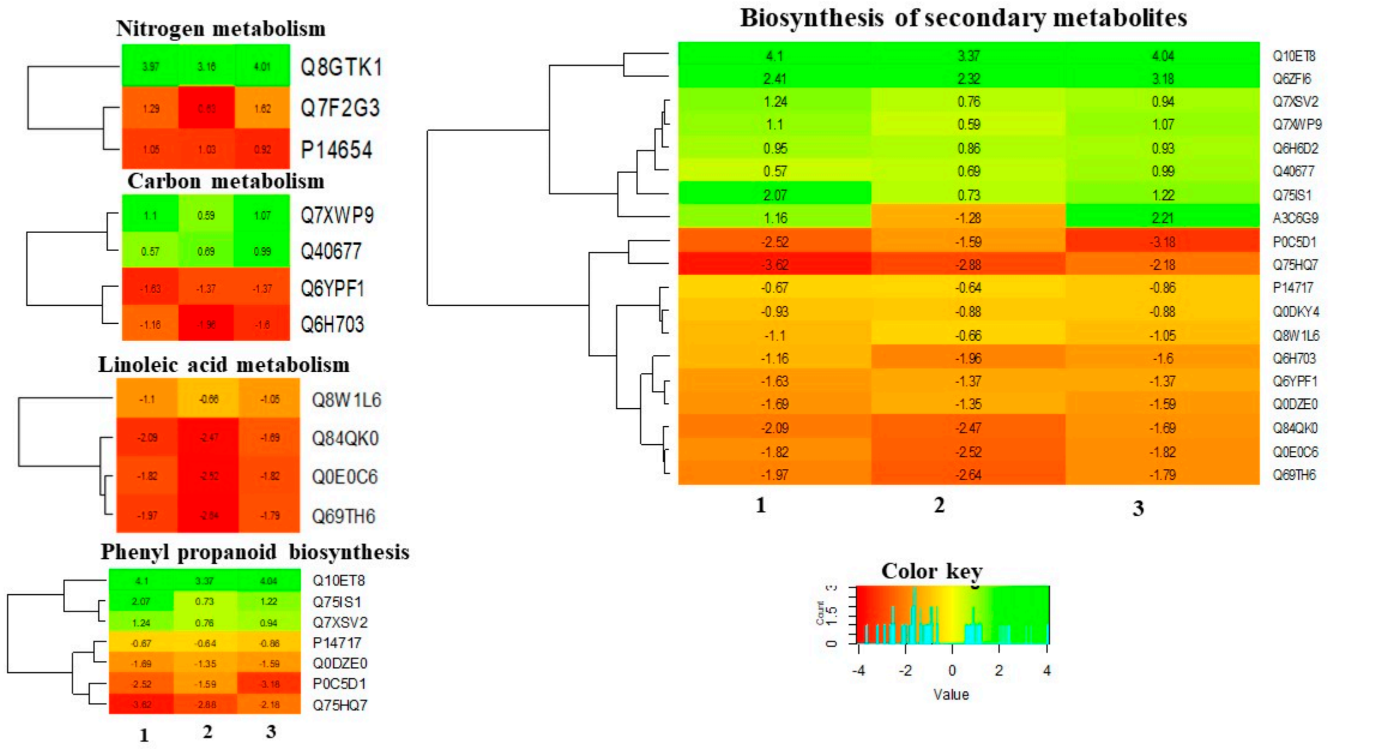

Supplementary Figure S3. Heatmap showing different metabolic pathways significantly (P value<0.05) enriched by 139 differentially abundant proteins (DAPs) present at all three-interactions (1. *O. sativa*+*P. indica; 2.O. sativa*+W5; 3. *O. sativa*+*P. indica*+W5). Green color represents fold change in upregulated proteins while downregulated proteins are represented in red. Proteins identified in different pathways are represented in their uniprot id's.

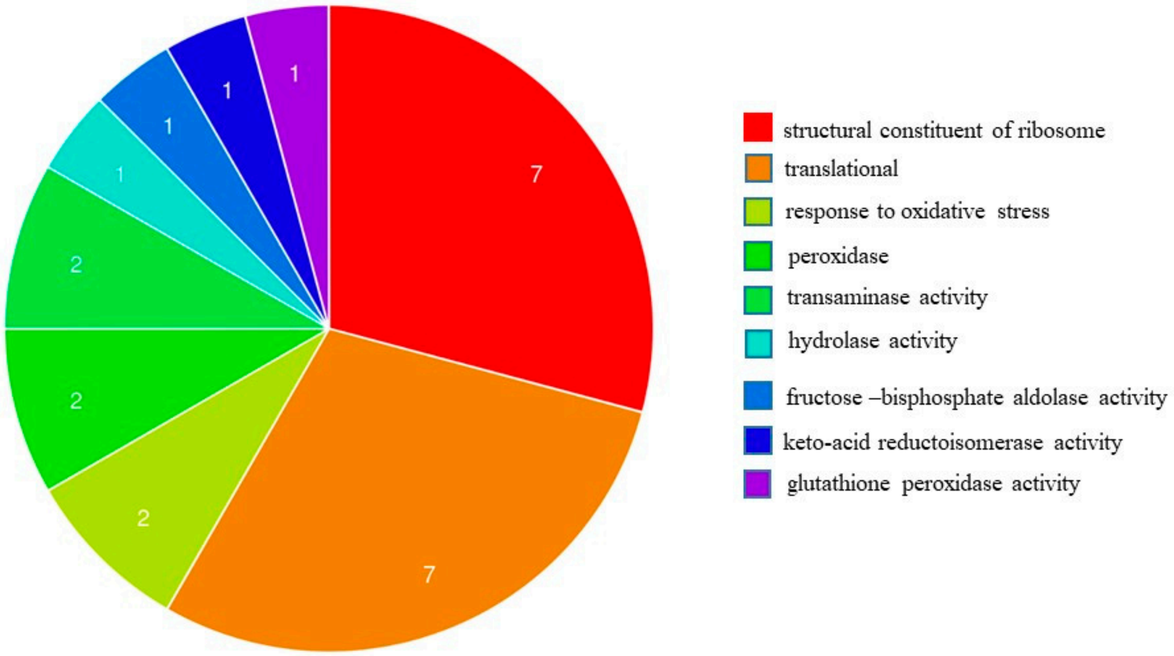

Supplementary Figure S4. Pie chart showing significantly (p value, P≤0.05) enriched biological processes and molecular functions for the genes corresponding to the *P. indica* DAPs, identified in the roots of the *O. sativa* in the presence of the W5. Numbers in the sectors represents number of proteins that were enriched to these biological process and molecular functions.
